# Unpacking collaborative depression care: examining two essential tasks for implementation

**DOI:** 10.1186/1748-5908-10-S1-A33

**Published:** 2015-08-20

**Authors:** Yuhua Bao, Benjamin G Druss, Hye-Young Jung, Ya-Fen Chan, Jurgen Unützer

**Affiliations:** 1 Department of Healthcare Policy and Research, Weill Cornell Medical College, New York, NY 10065, USA; 2 Department of Health Policy and Management, Emory University, Atlanta, GA 30322, USA; 3 Department of Psychiatry and Behavioral Sciences, University of Washington, Seattle, WA 98195, USA

## Objective

The Collaborative Care Model (CCM) is increasingly recognized as a best practice model to integrate evidence-based mental health care in primary care. Wide dissemination of the CCM, however, may be hindered by a lack of understanding of the relative importance of the different components of the model. This study aims to assess how fidelity to two key process-of-care tasks of the CCM predicts patient depression outcomes.

## Methods

We used registry data from the Washington State Mental Health Integration Program (MHIP), an implementation of the CCM among over 100 community health clinics. Fidelity to two key tasks in the early stage of CCM intervention was assessed: timely follow-up (>=1 follow-up contact with the care manager within 4 weeks of initial contact) and psychiatric consultation (>=1 consultation or evaluation within 8 weeks of initial contact among patients not achieving improvement by Week 8). Clinically significant improvement in depression was determined if patient achieved a 50% or more reduction in PHQ-9 score or a PHQ-9 below 10 within 6 months of initial contact. Logistic and Cox proportional hazard models were estimated to examine how fidelity with either task predicted improvement and time to improvement. All analysis was conducted with the original sample and with a propensity-score (PS) matched sample.

## Findings

Timely follow-up was associated with an odds ratio (OR) of 1.72 (95% CI: 1.46, 2.04) and a hazard ratio (HR) of 2.13 (1.88, 2.42) for achieving improvement in depression. Psychiatric consultation was associated with an OR of 1.11 (0.93, 1.33) and an HR of 1.13 (0.97, 1.32). PS-matched analysis yielded very similar results. Results suggest that timely follow-up in the early phase of CCM may significantly improve the trajectories of patient depression outcomes.

## Contribution to the field of D&I

Our study is among the firsts to unpack the CCM to examine the relative importance of different components of a complex intervention. Findings of this and future studies will inform resource-allocation decisions to maximize returns to implementation and the selection of quality assurance and performance measures for CCM implementation.

## Funding

National Institute of Mental Health. The MHIP registry data were originally collected for quality improvement purposes and were funded by Community Health Plan of Washington and Public Health of Seattle and King County.

